# Supplementary material for: Low-latitude zooplankton pigmentation plasticity in response to multiple threats
Source: R Soc Open Sci. 2019 Jul 24;6(7):190321. doi: 10.1098/rsos.190321 (PMC6689576; doi:10.1098/rsos.190321)
Supplement: Criteria for the Blue hole selection and raw data figure. [file rsos190321supp1.docx]

**Supplementary Material for Low-latitude zooplankton pigmentation plasticity in response to multiple threats.** Marcus Lee, Huan Zhang, Yongcui Sha, Alexander Hegg, Gustaf Ekelund Ugge, Jerker Vinterstare, Martin Skerlep, Varpu Pärssinen, Simon David Herzog, Caroline Björnerås, Raphael Gollnisch, Emma Johansson, Nan Hu, P. Anders Nilsson, Kaj Hulthén, Karin Rengefors, R. Brian Langerhans, Christer Brönmark and Lars-Anders Hansson**. *Royal Society Open Science***

**Additional Site Information:** We selected blue holes geographically near one another (all within a 3.7 km^2^ region) that all exhibit relatively similarly clear water. Based on 22 turbidity measurements taken with an Oakton T-100 turbidimeter (Vernon Hills, IL) in these blue holes from 2011 to 2018, sites range in turbidity from 0.55 NTU (Cousteau’s) to 0.65 NTU (Rainbow) to 0.68 NTU (Turtle), and do not significantly differ from each other (ANOVA: *F*_2,19_ = 0.54, *p* = 0.59). Average water transparency in these blue holes is 7.3 m based on Secchi disk measurements. Thus, UVR exposure is likely very similar across all three localities.

**Quantification of Predation Risk:** Temporally repeatable estimates of fish densities have been measured in these blue holes using underwater visual census (1, 2). Moreover, the total number of bites taken toward possible prey per minute during daylight hours for individual adult male and female Bahamas mosquitofish have also been estimated for these blue holes (3). Multiplying sex-specific feeding rates by sex-specific density, and assuming that potentially zooplanktivorous bigmouth sleepers (less than 10cm total length) take one bite per minute, we calculated an estimate of total daytime predation threat for small aquatic prey in each blue hole containing fish (Table S1). We recognise that, although Turtle has no fish predators, it likely has invertebrate predators which may hunt visually and therefore the true predation risk is greater than zero.

Table S1. Predation risk as quantified by observing the total number of bites toward possible prey by fish per minute per cubic meter during daylight hours.

| Blue hole | Predation risk min^-1^m^-3^ |
| --- | --- |
| Turtle | 0 |
| Cousteau’s | 11.59 |
| Rainbow | 35.61 |

Figure S1. Raw data displaying the variation in median pigmentation levels (Δa* = redness, Δb* = yellowness) of copepods (n = 25 per treatment X population) after exposure to the treatments in the laboratory experiment compared with the mean pigmentation of samples taken directly from the respective blue hole (hatched line). Note that the axes are different between the two pigments, yet the patterns are consistent.

References

1. Heinen JL, Coco MW, Marcuard MS, White DN, Peterson MN, Martin RA, et al. Environmental drivers of demographics, habitat use, and behavior during a post-Pleistocene radiation of Bahamas mosquitofish (Gambusia hubbsi). Evolutionary Ecology. 2013;27(5):971-91.

2. Martin RA, McGee MD, Langerhans RB. Predicting ecological and phenotypic differentiation in the wild: a case of piscivorous fish in a fishless environment. Biological Journal of the Linnean Society. 2015;114(3):588-607.

3. Heinen-Kay JL, Schmidt DA, Stafford AT, Costa MT, Peterson MN, Kern EMA, et al. Predicting multifarious behavioural divergence in the wild. Animal Behaviour. 2016;121:3-10.
